# Recycling of Plastics in the Automotive Sector and Methods of Removing Paint for Its Revalorization: A Critical Review

**DOI:** 10.3390/polym16213023

**Published:** 2024-10-28

**Authors:** Carla Zambrano, Pablo Tamarit, Ana Inés Fernandez, Camila Barreneche

**Affiliations:** 1 CITSALP, S.L., Calle Antonio Gaudí, 74, Nave 5 Pl. Ind. Rubí Sur., Rubí, 08191 Barcelona, Spain; czambrano@ub.edu (C.Z.); ptamarit@citsalp.com (P.T.); 2 Departament of Material Science and Physical Chemistry, Universitat de Barcelona, C/Martí i Franquès 1, 08028 Barcelona, Spain

**Keywords:** recycling, polymers, sustainability, automotive, paint removal

## Abstract

The presence of plastics in the automotive industry is increasingly significant due to their lightweight nature, which contributes to reducing fuel consumption and CO_2_ emissions while improving versatility and mechanical properties. Polypropylene (PP) and other polyolefins are among the most commonly used materials, especially for components such as bumpers. The use of composite materials, i.e., a combination of different polymers, improves the properties through synergistic effects, thereby also improving the performance of the final product. In the automotive industry, PP reinforced with 20% talc or CaCO_3_ is commonly used. The mechanical recycling of polypropylene bumpers is the most common type of recycling. However, challenges arise during this process, such as the presence of impurities like paint, chemical contaminants from previous use, and polymeric impurities from different polymers mixed into the polymer matrix, among others. Paint affects both the aesthetic quality and the mechanical and intrinsic properties of the recycled material. This review aims to analyze the main methods reported in the literature, focusing on those with low environmental impact. Furthermore, these methods are classified according to their capacity, effectiveness, substrate damage, environmental hazards, and economic feasibility. It also aims to offer a comprehensive overview of the mechanical recycling of plastic waste in the automotive industry.

## 1. Introduction

**Figure 1:**
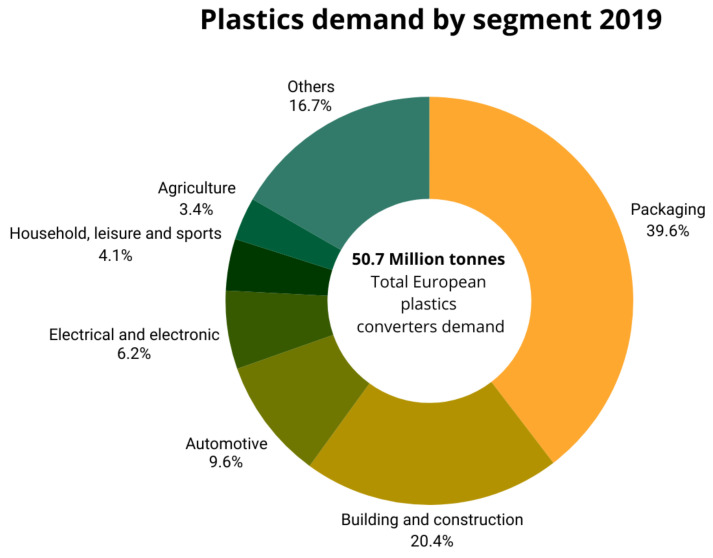
Demand for plastics according to sectors in 2019 [3].

The use of plastics in the manufacture of automobiles has been growing. Generally, metallic materials and alloys, especially steel, aluminum, and magnesium, have been the most commonly used materials throughout the history of automobile body manufacturing. The properties of steel alloys, such as mechanical resistance and hardness, make this material one of the most suitable candidates for this application. However, the great revolution of plastics in the automotive market began in 1950, when thermoplastics such as acrylonitrile butadiene styrene (ABS), polyamide (PA), polycarbonate (PC), and polymer composites began to take center stage in the development of plastics [1]. In 1956, plastic was used for the first time in the bodywork of automobiles. The lightness offered by these polymers to the total weight of the car leads to a decrease in fuel consumption by 12 tons per year and reduces CO_2_ emissions by 30 times [2]. Plastics also offer additional advantages over other materials. Its great tenacity reduces the damage in low-speed blows, and its malleability reduces the complexity of the assembly, reducing costs in this production line. Resistance to chemical agents due to structural stability, low electrical and thermal conductivity, good resistance to corrosion and degradation, good optical properties, and the low cost of most commercial polymers are some of the many benefits that plastics present compared to other materials. Moreover, the extensive range of polymers available on the market and their diverse properties make plastics remarkably versatile. This versatility enables their use in various parts of the car, both in the interior and exterior. Thus, the use of synthetic materials such as plastics has increased in the automotive industry in recent years, making them one of the main materials in many vehicles; up to 50% of the weight of some vehicles is plastic. Among all plastic applications, the automotive industry is the sector with the third highest demand in Europe, with 9.6% in 2019 (Figure 1), representing an increase of more than 1% since 2013.

The main polymers used in the automotive industry are thermoplastics, elastomers, and thermosets. Among the well-known thermoplastics are ABS, PA, PC, polypropylene (PP), polyethylene (PE), and polyvinyl chloride (PVC); sometimes, combinations between these or with elastomers such as PP-EPDM (polypropylene–ethylene propylene diene monomer), ABS-PC, and PC-PBT (polycarbonate–polybutylene terephthalate) are also used. Thermoset composites like fiberglass-reinforced polyester and thermosets like epoxy resins, as well as elastomers such as polyurethane, are primarily utilized for interior coatings [4]. PCs are usually used in non-reinforced conditions, and their applications could be for bumpers, headlamp lenses, and security screens, among others. ABS, polyphenyl ether (PPE), PC, and PP are used in dashboards.

**Figure 2:**
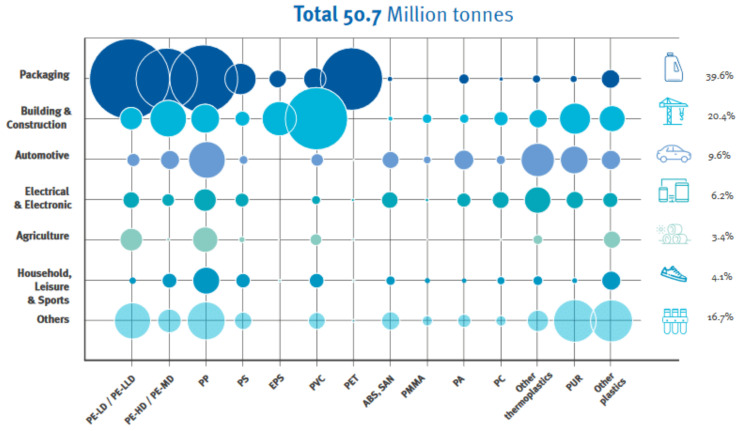
Distribution of plastic demand in Europe by polymer type and industry. Courtesy of PlasticsEurope [3].

In a midsized car, more than 1000 parts of different shapes are made of polymers [1]. Nevertheless, three types of plastics—PP, PE, and polyurethane (PU)—represent more than half of these materials used in the automotive industry (Figure 2). The projected growth in the use of these and other polymers in the automotive sector is also on the rise [5]. PP is the most commonly used plastic in the automotive sector [6]. It is used for front and rear bumpers, chemical tanks, cable insulation, and even carpet fibers.

### The Use of Composites in the Automotive Sector

**Figure 3:**
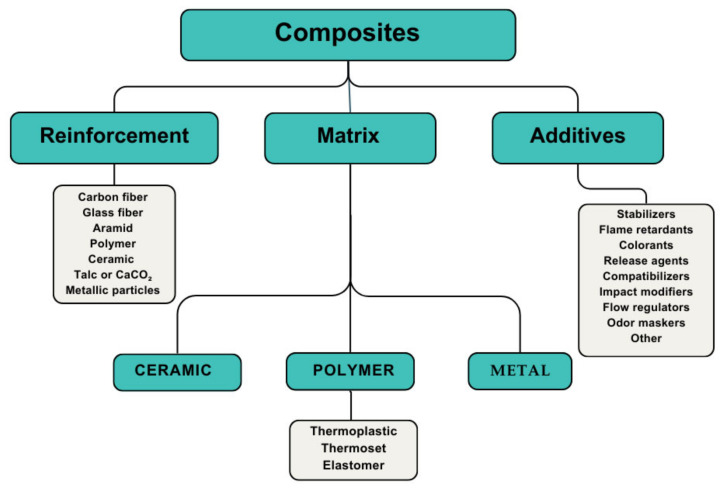
Most common constituents of composites.

Composite materials are increasingly being used in the automotive industry due to their excellent strength-to-weight ratio and the modifications they bring to the matrix [7,8]. The combination of two or more different compounds forming composites gives a synergistic effect, improving the properties they have separately. Composites with a polymeric matrix are generally made up of a reinforcement, a matrix, and additives (Figure 3).

In the automotive sector, in the absence of fiberglass and carbon fiber, talc or CaCO_3_ are widely used as reinforcements for their lower price [9]. It is very common to find PP reinforced with 20–30% talc, CaCO_3_, or rubber (e.g., EPDM) in the bumpers of many cars [10]. Several researchers have reported the strong influence of different compounded talc or rubber on the microstructure and mechanical behavior [11,12]. PP filled with talc increases the tensile modulus; however, the yield strength and Izod impact resistance decrease. Conversely, PP filled with rubber enhances the impact strength and elongation at break but leads to a decrease in both yield strength and bending strength [13].

The use of reinforced plastics affects the machining processes, as the thermal and mechanical properties of each of the components of the composites are different. Therefore, factors such as cutting speed and depth of pass must be considered because they could produce an absorption of the polymer matrix and result in alterations to the mechanical properties. It is recommended, among other considerations, that the machining process be carried out without cooling [7]. Figure 3 shows a scheme of the most common constituents of composites.

## 2. Bumpers on Cars

**Figure 4:**
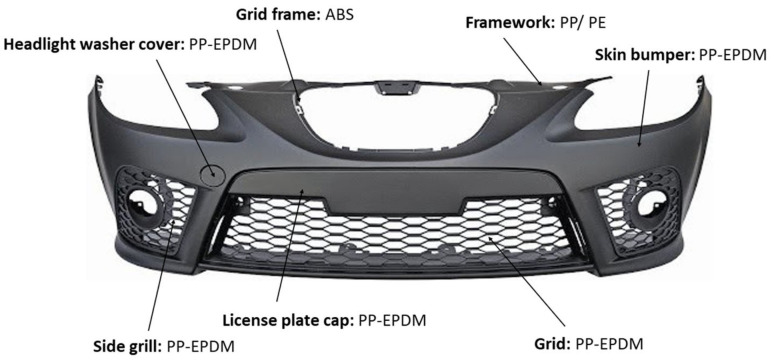
Front bumper Seat Leon 04-09 Look FR/Cupra Plastic with the type of plastic for each part.

The main mission of the bumper is to cushion and protect the vehicle in the event of a collision. This is achieved through the absorption of kinetic energy, mitigating potential damages that may have arisen in the absence of the said safety component [14]. The bumper represents approximately 9 kg of the total weight of the vehicle. Therefore, the material from which this very important part of the vehicle is made must be deformable to cushion the impact (Figure 4). The first use of plastics inside cars was in the bumper, which was completely redesigned in the 70s thanks to the development of polymers. According to a study on the composition of bumpers of different brands and models [15], they are made of 91% polyolefins (PP and PE). The introduction of PE into PP is commonly carried out to increase the impact resistance and improve material properties. Although the compatibility of PP and PE is not good, compatibilizers are added to make mixing possible. These additives reduce the interfacial tension between them and improve the interaction. They can also be recycled together as copolymers thanks to polymer compatibility aids. On the other hand, EPDM is also usually added as a reinforcement. It is an elastomer with very good properties against atmospheric agents, with high elasticity and resistance. It also takes advantage of its function as a compatibilizing additive between PP and PE. In Europe, bumpers are mainly made of polypropylene but can also contain metal inserts (chrome or aluminum). Other materials used in the manufacture of bumpers are PU, ABS, acrylonitrile–styrene–acrylate (ASA), and PC with polybutylene terephthalate (PC/PBT), but they are less used than PP.

Composites are one of the main materials used in the manufacture of bodywork, especially bumpers. Some of the advantages of using glass fibers, talc, and other reinforcement are the excellent adhesion between the fiber and the matrix, the good mechanical resistance, fireproofness, good resistance to chemical agents, easy processing, and good dielectric properties, among many others [7]. However, the presence of mineral fillers in the material increases the density of the material, making a heavier bumper piece.

### Lightweight in Cars

The functional and environmental requirements of the automotive sector are becoming more stringent due to the increase in pollution and the need to improve fuel economy [16,17]. However, since 2007, after an increase of almost 50% in CO_2_-equivalent emissions, there has been a decrease in emissions due to the economic crisis and the effectiveness of measures imposed on the sector. The European Union in 2014 set greenhouse gas (GHG) emission targets for 2020 of 95 g CO_2_/km for new cars sold. Manufacturers will need to reduce average emissions from their new vehicle sales by 15% by 2025 (from 2021 levels) and by 37.5% by 2030 [18]. The European Green Deal committed to reducing GHG emissions by 90% by 2050 compared to 1990, with transport accounting for a quarter of all GHG emissions in the European Union [19]. Numerous studies have discussed the relationship between weight reduction and improved fuel efficiency. Therefore, the use of materials lighter than conventional ones has increased considerably for the manufacture of automobiles. In 2016, Pervaiz et al. from Canada suggested that the European commitment to reduce CO_2_/km to 95 g by 2020 requires a reduction in vehicle weight of about 200–300 kg [17]. In the same year, the National Academy of Science, Engineering, and Medicine, along with other contributors, published an article where it claimed that reducing weight in the vehicle can reduce the rolling resistance of the tires, which is proportional to the weight of the vehicle [20]. It also reduces the amount of energy required to accelerate a vehicle and thus the amount of energy lost by brakes [20]. They described that the average engine efficiency would be affected by the duty cycle and that there was contribution from main elements such as aerodynamics, weight, and tires. In 2020, Kotaro Kawajiri et al. analyzed the impacts of replacement materials on fuel efficiency and GHG emissions [21]. They concluded that weight reduction was imperative, but, interestingly, while manufacturers were increasingly using lightweight materials, the weight of the vehicle had increased due to “design bounce” from the addition of features and performance as vehicle generations progressed. In addition, we have to take into account not only the reduction in emissions with the use of lighter materials in manufacturing but also the carbon footprint of the entire life cycle of the manufacture of such materials [21,22].

This review aims to highlight the existing developments in the literature to help solve some of the problems that prevail in the process of recycling plastics used in automobiles, especially in the elimination of pollutants such as paint. An evaluation of conventional recycling processes is also provided, focusing on polymeric waste from the automotive sector and polypropylene and polyolefins used to manufacture front and rear bumpers. The processes used in mechanical recycling are discussed, giving a review of the types of recycling and thermoplastics that are mostly incorporated in the plastic recycling system.

In addition, this review aims to inspire innovation to achieve higher-quality recycling production from mechanical recycling of plastics, including reuse and revaluation, thus creating the economic and environmental boost necessary for a circular economy.

## 3. Circular Economy

**Figure 5:**
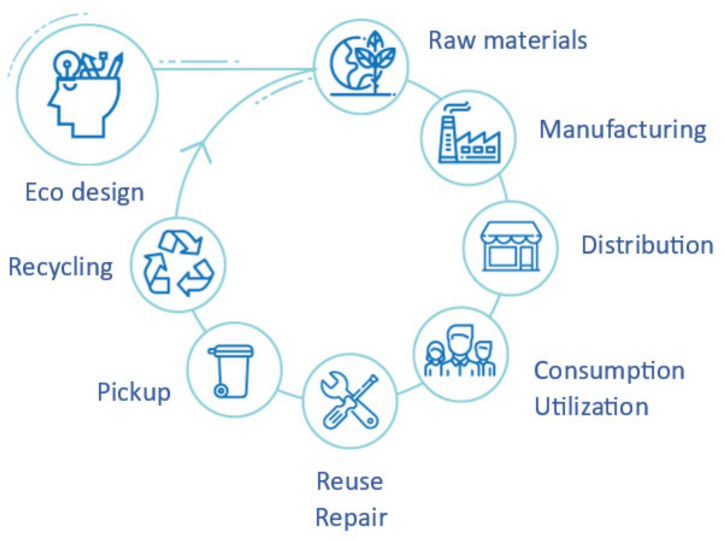
Step diagram for a circular economy [26].

The realm of recycling, particularly when it comes to plastics, amplifies the significance of embracing a circular economy by adding value. After all, within a good circular economy system, it is necessary to maintain the value of products, materials, and resources for as long as possible [23]. In this way, it is possible to minimize the generation of waste and avoid indirect problems that create a network of bad economic and environmental practices. A good scheme of what the circular economy is based on is shown in Figure 5. Good functioning of this closed circle of production and consumption will require increased association between consumers, buyers, and the desirable function that a given product provides, not only the product itself [24]. In an economic model that meets these requirements, there should be a clear economic incentive to reduce the use of resources and increase the efficiency and longevity of the product [25]. It should also be committed to the elimination of waste through innovation, the development of reuse and recycling opportunities, and possible improvements throughout the transformation process. Collective responsibility and the ability to reinvent and/or seek alternatives to the accumulation of waste and the production of non-recyclable materials by large companies is one of the most discussed challenges in the literature.

The promotion of the circular economy represents a gain for many parts of the production–consumption cycle. Plastic producers win by saving in waste management, recycling companies by increased recycling, manufacturers of auto parts by reducing their cost for raw materials, and technology and sustainable innovation centers by gaining capital to carry out projects that focus on the development of technologies that facilitate the recycling process. In addition, the planet and the environment avoid a significant accumulation of almost permanent waste and a depletion of resources that are not unlimited. In conclusion, the circular economy is the answer to more sustainable world development.

## 4. Recycling of Plastics in Automobiles

The properties of plastics make them very interesting candidates in the development of materials for the automotive industry. Nevertheless, plastics have important drawbacks that create environmental problems when mass manufacturing of this material occurs. These drawbacks include the large volume, which creates storage and space problems in the short and long term. Its time of permanence on the planet can exceed 400 years, and many of the plastics that are used cannot be recycled. In addition, many of the microplastics that are not trapped in the filters of the treatment plants represent waste for the marine fauna, which causes alterations in the feeding and reproduction patterns. These and other disadvantages have made the plastic recycling and recovery sector grow exponentially year after year in recent decades [27]. However, the replacement of plastics is difficult due to their good performance and the increase in CO_2_ emissions from the transport of other heavier materials. Therefore, the recycling and reuse of plastic is one of the alternatives to solve these problems.

**Figure 6:**
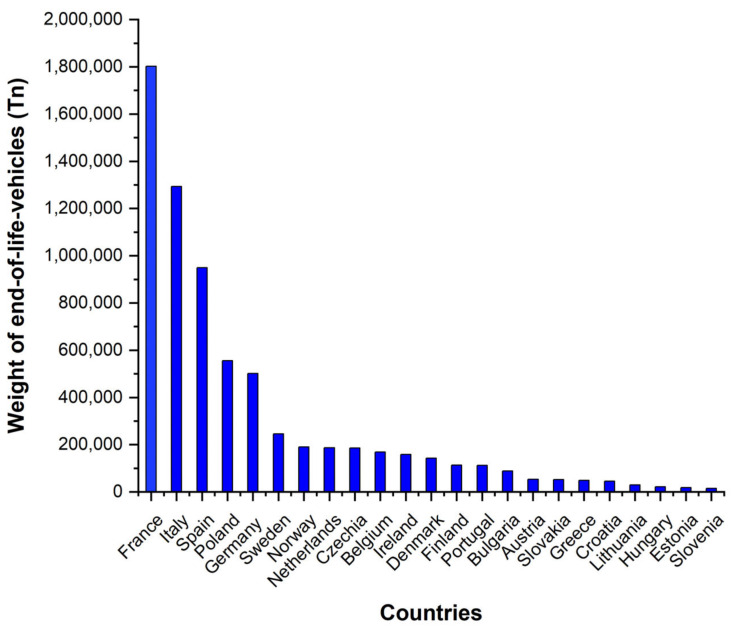
Recycling of ELVs in 2019 in tons of material for each country [28] (own elaboration).

From the legislative point of view, both in the European and national scenarios, recycling is being promoted, forcing compliance with requirements that aim to increase both the use of recycling and the manufacture of recyclable materials. In the automotive industry, according to the European directive 2000/53/EC, since 2015, manufacturers are obliged to reuse 95% of the average weight per vehicle and year: 85% recycled and 10% in general energy. Reuse and recovery rates for most EU members exceeded 85% in 2009 [28]. The countries with the highest contribution to recycling end-of-life vehicles (ELVs) are France, Italy, Spain, Poland, and Germany (see Figure 6).

**Figure 7:**
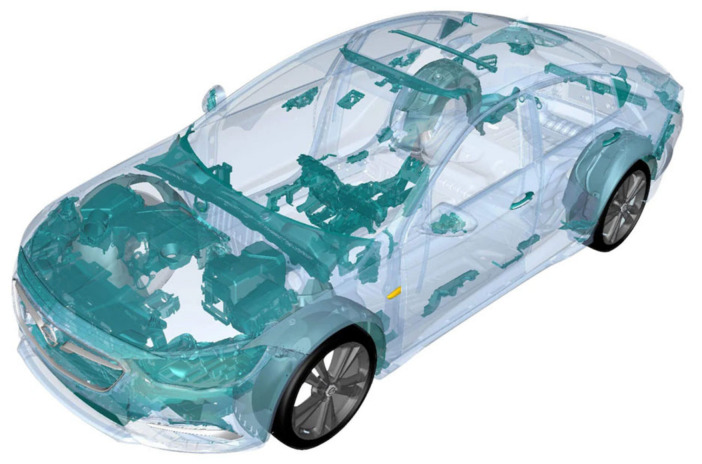
Recycled plastic parts in the Opel Insignia vehicle [32].

The automotive sector probably has the best recycling record registered [29]. Fortunately, many automobile manufacturers are joining the initiative to use recycled plastics in their manufacturing designs by increasing the percentage of recyclables in new vehicles. In fact, the Renault Group started using recycled plastic in its vehicles in the 1990s [30]. Ford uses recycled plastics for passenger seat upholstery, using more than 50 million pounds of plastic in numerous models like the famous Focus [29]. Opel is also committed to meeting recycling targets in “recycling and recovery” guidelines, and they try to ensure that the quality of their treatment processes allows them to use these materials for visible components of their vehicles (Figure 7). Honda recycles bumper waste generated during the manufacturing process, which is reformulated and reused in Honda’s supply chain to make splash and mud guards [29]. The commitment of some brands, such as Volvo, which ensures that by 2025 at least 25% of the plastics used to manufacture their vehicles will be recycled as part of an anti-pollution plan praised by the United Nations, augurs a good future for the sector of plastic recycling [31]. In the redesigned Clio IV (2016), recycled plastic is the main material used in bumpers and wheel archliners.

**Figure 8:**
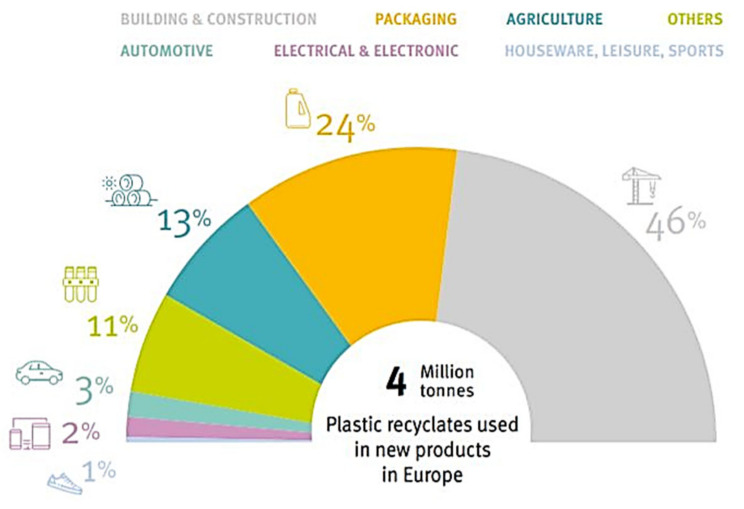
Use of recycled plastics by sector in 2018. Courtesy of PlasticsEurope [3].

However, the inner lining, which represents almost 40 kg of plastic, accounts for a very small fraction of the overall recycling; therefore, there is still much room for improvement in the recovery of plastics and in the development of technical polymers from recycled materials [15]. The protagonist in the use of recycled materials is the construction sector. In 2018, 3% of plastic recyclates were used by the automotive sector (Figure 8). Consequently, in most cases, there is no closed recycling cycle within the sector.

Currently, there are different recycling processes that are essentially divided into mechanical, chemical, and thermal. The subject will not be delved into as it is not the main objective of this work, but what each of them consists of will be briefly explained.

**Figure 9:**
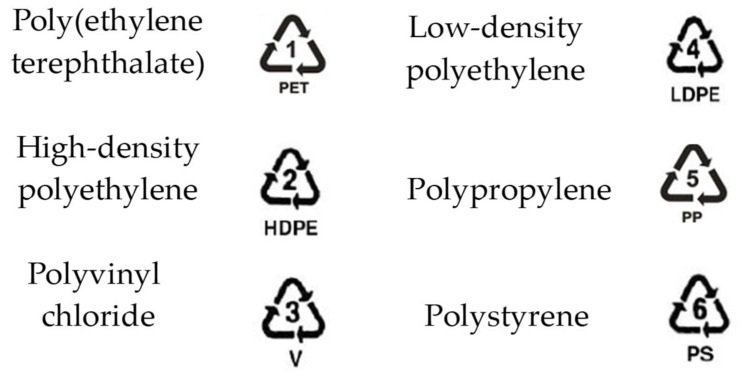
Commodities thermoplastic classification.

The first step in recycling is the classification of the different materials that arrive from their end of life of application. It is aimed at processing each type of plastic with the required specifications and avoiding contamination from others within the process. This classification occurs with optical or flotation methods due to the density changes in the different polymers. The most common plastics in recycling are some of the thermoplastics that enter the recycling classification (see Figure 9).

Apart from these thermoplastics, there are also ABS, SAN, PA, PC, polymethylmethacrylate (PMMA), polyarylsulfone (PSU), fluoropolymers, polyetheretherketone (PEEK), polyacetal (POM) and PBT, among others.

Mechanical recycling consists of processing the material from consumption using pressure and heat to reshape it, allowing it to be used later. They are based on extrusion, injection, blowing, compression, or thermoforming processes. This recycling route is possible with materials that can be melted and cooled back to room temperature with minimal changes to their properties, such as thermoplastics. However, sometimes, a degradation of material properties occurs due to recycling cycles that bring the material to melting temperatures. This thermal degradation contributes to the weakening of the intermolecular forces of the polymer chains. One of the effects of this degradation by repeated recycling of polymers linked to chain scission was found to be the reduction in crystallinity [33].

In chemical recycling, macromolecules break down as a result of catalysts or heat treatments, allowing monomers of the said polymer to later obtain new plastics. These treatments induce depolymerization through processes such as pyrolysis, hydrogenation, and thermal cracking; dissolution; or solvolysis, such as hydrolysis, methanolysis, and glycolysis (with ethylene glycol) [34]. This mechanism is useful for plastics that cannot be mechanically recycled as multilayer plastics or with a high level of environmental degradation.

Thermal recycling, also known as energy recovery, aims to convert polymers into fossil fuels or raw materials for high-temperature furnace manufacturing processes of other materials. This process capitalizes on the high heat capacity of polymers, which is approximately 67 J·mol^−1^·K^−1^ in the case of PP [35]. This type of recycling is recommended for highly deteriorated materials or for those mixed with other materials that are challenging to separate.

According to a recent study on the environmental impact of the cycle of chemical recycling of plastics in comparison with mechanical and energy recovery, chemical recycling showed a greater impact on climate change than mechanical recycling but 42% less energy recovery [36]. However, the recycling route must be studied because there are differences if pyrolysis, acidification, or eutrophication are chosen as forms of chemical recycling. In Europe, mechanical recycling is the most commonly used after the energy recovery of plastics [37].

### 4.1. Recycling of Polypropylene Bumper

Polypropylene has a great presence in the automotive sector. It can be presented in three different forms: homopolymer, random copolymer, and impact copolymer. The first is composed mainly of polypropylene, the second can contain traces of ethylene from 1 to 8%, and the third can contain ethylene levels of 45 to 65%. The different proportions of ethylene cause its properties to change [6]. The homopolymer is stiffer than the other two; therefore, it is less resistant to impact and breaks more easily. Polymer forms that contain some traces of ethylene are generally more flexible and have more impact, improving the quality of the material for the automotive sector. PP is one of the lightest thermoplastics due to its low density of around 0.9 g/mL used in both interior trims, such as doors and consoles, and exterior parts, such as bumpers [6]. Therefore, we can assert that polypropylene is the most crucial material within the polyolefin family due to various reasons: possessing good properties such as high melting temperature, chemical resistance, low cost, and low density; being a very versatile material; and offering the possibility of different morphologies using fillers and creating blends of polymers with PP to achieve superior characteristics.

The amount of plastic used in both front and rear bumpers (10 kg/car for small to medium cars and 13 kg/car for sports utility vehicles (SUV)) was approximately 172,000 Tn for 15.1 million new cars registered in 2018 [38]. This is a very large volume, highlighting the importance of recycling vehicles at their end of life. Recycling of polypropylene as a thermoplastic polymer is possible because it melts when it reaches a melting temperature and becomes hard and brittle when it cools, meaning it does not significantly lose any of its properties. Therefore, its recycling is much easier than other types of plastics.

**Figure 10:**
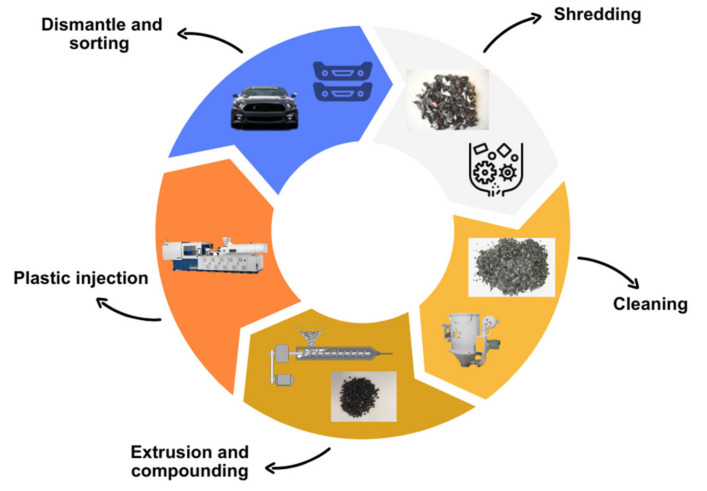
Scheme of the recycling process of bumpers (own elaboration).

The main recycling process used for PP in the automotive sector, especially for front and rear bumpers, is a mechanical process through extrusion and injection. However, the process is more extensive and can be divided into several phases (Figure 10):

Dismantling of the bumper part of the whole car (or any part to be recycled).
Sorting and separation of different materials according to the type of plastic.
Separation of contaminants such as paper and dust in a cyclone.
Separation of other recyclable polymers by means of a floating tank due to the differences in density.
Shredding and turning large pieces of plastic into small ones.
Washing and drying to clean the plastic, usually with water or a chemical such as caustic soda or surfactants.
Extrusion to form pellets.
Agglutinating involving additivation to improve some properties that have decreased with the processing and use of the material or the introduction of fillers.
Cooling by letting the material stand at room temperature or heat drying if it has moisture.

Usually, the order of the steps varies according to the origin of the waste, its composition, and the way of working of the recycling entity. Through mechanical recycling, an optimal material can be obtained with properties as similar as possible to the virgin material for use in the same, similar, or totally different applications. The cost of raw materials and fluctuations in the market affect the price of recycled materials. The price of these should be closely related to the quality of their chemical, mechanical, and thermal properties. However, these materials are only preferred when they cost the same or less than the virgin material. After all, these materials have already been used, and the quality compared to their virgin analog will be generally lower. Therefore, the economic factors that influence the viability of recycling thermoplastics, such as PP, are the price of the recycled polymer, the virgin polymer, the recycling process, and the alternative materials for that application [39].

The requirements for recycled materials depend to a greater extent on the application or their destination, so the problems that may arise also depend on it. For visible materials inside the car, the color and texture, such as the smell and the emission of volatile compounds (VOC), should have controlled characteristics. However, for exterior parts, the obstacles are more related to the paint and its durability, mechanical properties, and impact resistance. The importance of developing and optimizing recycling channels could provide a solution to the problems that prevent greater reuse of recycled material in percentage by weight of final products.

### 4.2. Additivation

**Figure 11:**
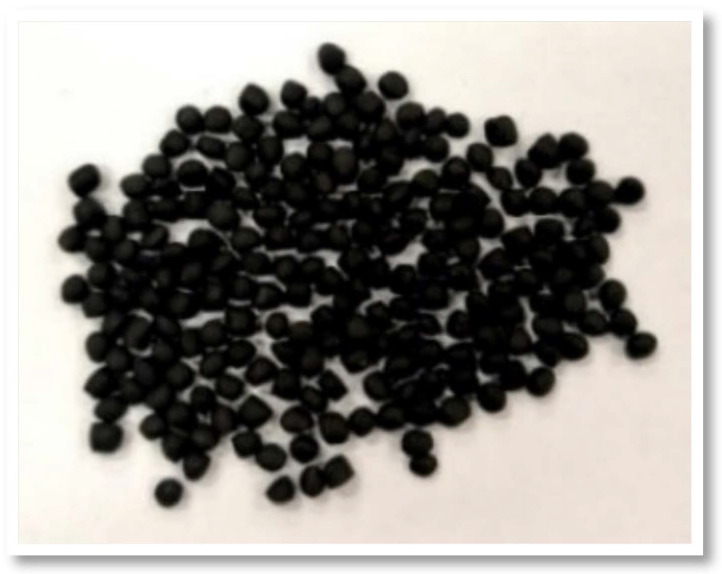
Black masterbatch for processing recycled PP pellets (own elaboration).

Additives are present in the polymer synthesis process to help stabilize the final product or provide them with other properties, such as achieving pigment dispersion, gloss, or agglomeration, among others. Therefore, in the plastic recycling process, an additive becomes a very important reinforcement as it consolidates the quality of the polymer and allows the recycled material to be used in applications that would not have been possible without this additive [11]. There are multiple types or additive families that eliminate or mitigate many of the problems that arise in the process, such as poor stabilization, low mechanical properties, possible odors depending on the origin of the material, contamination with third parties, colors that indicate the principle of degradation, inadequate fluidity, etc. Stabilizers like antioxidants, thermal stabilizers, and ultraviolet (UV) stabilizers are generally added to recycling as they have mainly been consumed during the first shelf life and in plastic processing [40]. It is also common to use impact modifiers, crosslinking agents, compatibilizers, pigments, flow regulators, bleaching agents, and plasticizers, among many others. One of the most widely used additives, thanks to its dilator and filler function, is CaCO_3_ or talc, which provides greater rigidity to the material and resistance to tension. Carbon black is also commonly used as a filler and pigment, antistatic agent, or auxiliary to form cross-links. Recycled plastic is normally distributed in the form of pellets after its extrusion and the introduction of carbon black in the form of masterbatch, known as black master (Figure 11). It consists of concentrated pigment dispersions incorporated in a resin base, normally the same as that of the resin to be colored or in a support that is compatible with the material.

It also depends on the form in which the polymer is presented and the nature of its composition; the addition of these additives can be achieved in several ways. Polypropylene, for example, if present in powder form, is homogeneously mixed with pigment powder preparations and stabilizers. When it is presented in the form of pellets, it can be mixed dry by dosing the components in the extruder, in the injector, or by pre-extrusion of the components [41]. They can be added in the final process or in the compound mixing stage.

Generally, special extruders are used for compounding, presenting screws with a mixing zone for better homogenization of the material with the filler or additive or, failing that, twin screw extruders. The additives must be compatible with each other in the case of coexistence between them in the same polymeric matrix or between the matrix and the additive. Some attention must be paid to this stage as injection problems could arise.

## 5. Challenges in the Recycling Process

The process of mechanical recycling of polymers has been developed over the years until reaching the point where it is currently possible to recycle thermoplastics and even some thermosets by spraying them to reuse them as fillers. Reducing the degradation that exists in the reprocessing of already recycled materials due to the use of additives is possibly one of the most valuable lines of research for the plastic recycling cycle. Duval and MacLean state that the main problems with recycled plastics are based on limitations to market applications, the low value of recycled resin, and concerns about its effectiveness [42]. All of these limitations share a common denominator: the possible low quality of the recycled material. These technical restrictions affect both the classification of the materials and the presence of visible impurities present in the initial application of the material, such as adhesives or paints. The main cause of this problem is the difficulty in separating impurities or contaminants, such as other incompatible materials that are present in many recyclable polymers, which reduces their value and quality and greatly limits the range of applicability of these materials.

Solutions to these process problems will give the opportunity to create a closed recycling cycle in the automotive sector and increase the recovery of contaminated plastics in percentage by weight. Some materials arrive painted in different colors and with paints of various origins and compositions for the recycling process. The randomness and technical ignorance of these impurities due to their recycled nature adds to the difficulty of finding solutions to reach the purification of the material.

### Effect of Paint on the Quality of Recycled Material

The presence of paint in the recycling process presents difficulties in the initial sorting process if the painted plastic is present in a polymer blend or other properties of paint interfere with the process. The mechanical properties of recycled materials can be compromised by the presence of paint particles due to the stress concentrations created by them [34,43]. In addition, these impurities can be unstable in the extrusion process, as they produce gases or decompose the material in more complex situations. The amount of paint affects the rheology of the polymer in the sense that it produces defects in the injected part after its viscous state at high temperature. Therefore, when it is time to inject the polymeric fluid, it does not fill the mold in the same way if it has many paint particles. Paint can affect the strength and toughness of plastics through chemical, thermal, and mechanical interactions [44]. It can also affect the impact of the material and its elongation at break [45,46]. These effects can be found in both recycled materials and virgin materials. That is why it is so important to choose the best paint system and the right adhesion promoter for each situation to ensure the durability of the painted material [47]. Even if the painting system was compatible with the substrate at the beginning of its useful life and had been useful for many years, many of the properties can be compromised by the presence of this paint once it has been processed for recycling.

To solve this problem and eliminate this paint without damaging the polymer matrix, various paint stripping methods for thermoplastic polymers have been used over the years. Many of the authors who have developed these methods have classified them as mechanical, thermal, or chemical. The mechanical methods are mainly based on the impact of other particles on the paint film, inducing friction or crushing the painted material to a smaller particle size, among others. Thermal methods use the increase in temperature to induce softening of the paint layer and allow them to be removed later, for example, with a filter. Finally, chemical methods generally use organic solvents with an abrasive nature capable of penetrating the paint film to remove it without damaging the polymeric substrate.

## 6. Bumper Coating Removal Technologies

Most of the methods that have been found in this state of the art are based on a mixture of chemical, thermal, and mechanical methods. The classification can also be carried out by the appearance of the material to be treated with this method. Many methods use the crushed material with paint to introduce it into the paint stripping system. Others use the entire piece of plastic, such as an entire bumper. However, none of the methods treat the material in the form of pellets, as it normally presents carbon black masterbatch that, in many cases, covers the paint chips on its surface and makes the stripping process difficult. This makes the process economically less viable due to the addition of the extrusion and grinding processes. Problems are also attributed to the presence of paint within the intrinsic body of the pellets. However, treatments can be found for other substrates, such as wood, which can be studied in the future if they have functionality with polypropylene bumpers.

An investigation of the developments by different authors over time also shows several registered patents on stripping methods with engineering innovations and flow diagrams that give an understanding of the process [48,49,50,51,52,53,54]. Typically, these patents use chemical treatments based on benzyl alcohol or NMP (N-methyl-2-pyrrolidine) in an alkaline environment or surfactants and flocculants. In this essay, we have tried to chronologically order the methods found in scientific papers to observe the complexity of these and present an organization criterion (Table 1).

The approach required to remove the paint coating depends on the nature of the paint and its thickness, the substrate, and the interaction between them. Paint has several components, such as binders or resins, pigments, fillers, solvents, and additives. Within each of them, there are different types, such as acrylic, alkyd, amine, epoxy, and polyurethane resins, among others. In the automotive sector, the most common resins for the manufacture of exterior paint on bumpers are acrylic, polyurethane, epoxy, and amine [55]. The type of binder determines the specific levels of adhesion, sealing, gloss, and protection, as well as its way of drying and final curing. Therefore, to select the paint removal method, the constitution of the components to be treated must be known because there is no universal technique that is optimal for all combinations.

**Table 1:** Description of technologies developed for paint removal in polymers.

Reference	Summary
Ikai et al. 1994 [56]	Hydrolysis in an autoclave at 150 and 160 °C and 0.48 and 0.8 MPa, respectively, and subsequent dehydration with a centrifuge.
Handa 1996 [57]	Paint identification system with specific dyes and a hydrolysis method of extrusion for the removal of paint.
Ohori et al. 1996 [58]	Introduction of a heterocyclic azole-type modifier that acts on the ether bond of resins such as melamine acrylics.
Quazi et al. 1998 [59]	Use of filters with microscopic mesh size by melting at 220 °C.
Mizutani et al. 2000 [60]	A process of beating with blades in an aqueous environment at 130 °C.
Weston et al. 2005 [61]	Elimination of polypropylene paint with solid CO2 cryogenic particles at −78.5 °C at 190 m/s and their impact on the substrate.
Shipway et al. 2007 [62]	The impact of solid silica particles at 1.6 g/s in polypropylene mixtures used for bumpers.
Nitta et al. 2013 [63]	Paint removal from shredded bumpers using a whipping process and a subsequent optical method to optimize the stripping rate.
Mizutani 2014 [64]	Friction method between shredded pieces of painted bumpers and application of pressure at the outlet of the beating system with temperature.
Zhang et al. 2015 [65]	Theoretical and experimental study on the removal of coatings and paints with water jet technology.
Carlsson 2015 [66]	Synthesis of microemulsions with surfactants and additives to create a paint stripper.
Sarwono et al. 2018 [67]	Ionic liquids as an alternative to volatile organic solvents for the removal of alkyd paint on wood substrates.
Sover 2019 [68]	Removal of paint on thermoplastic substrates such as polypropylene by laser and determination of the influence of the laser on the surface of the polymer.

## 7. Discussion

Table 2 summarizes all paint removal methods mentioned in this revision of the state of the art.

The economic feasibility of methods using micron-sized filters depends on the amount of material to be stripped. The filter becomes very dirty when a coarse amount of material passes through, and multiple filters are required for effective removal. However, the elimination is not 100%. Therefore, the paint elimination is not optimal.

There are several methods based on high temperature in an aqueous environment found in the literature [56,60]. The water used in these methods can hydrolyze many coatings due to its polar nature and its good interaction with several types of paint resins. However, some plastics can be susceptible to degradation when in contact with water (especially condensation polymers). Therefore, careful control of the processing conditions is required when the polymers are working under these types of procedures [56,57]. Polypropylene is an apolar polymer and has resistance to water, like other chemical agents. This makes the degradation problem disappear a priori. Then, the removal effectiveness depends on the test conditions.

Otherwise, the methods that use organic solvents or other chemical substances share the problem regarding the management of toxic waste, which can be harmful to the environment and to the operator. In addition, these solvents can be a problem for the polymeric substrate, as many polymers are soluble in aromatic or chlorinated organic solvents, such as polypropylene.

Mechanisms that present friction between small pieces of painted bumpers have shown that a rise in temperature is created inside the container [60,64]. This increase in temperature causes the resistance to peel between the paint and the substrate to gradually decrease, and these collisions help to peel off the paint. However, the conditions of friction, speed, temperature control, and painting conditions need to be considered in order to consolidate its effectiveness.

Methods involving blasting of solid particles, cryogenic techniques, and high-pressure water are proper alternatives. The type of particle used in each case needs to be taken into account for blasting methods. For example, tests with painted polypropylene and glass ball particles have shown that paint removal is inefficient [62]. In addition, the use of dry ice as the impact particle is an environmentally friendly method, but paint removal is not properly achieved [61]. In general, these types of methods are used with the entire piece, and this reduces the economic feasibility if a large amount of material needs to be processed. The damage to the substrate should also be studied depending on its hardness and susceptibility to colliding particles.

On the other hand, some studies have been developed using thermal methods such as laser technology, optimizing the conditions of intensity and frequency of the beam for optimal results of paint removal without damaging the plastic substrate [68,69,70]. However, the cost of this technology is higher than other, more conventional methods, and it would increase the final value of the recycled material, being more expensive than the virgin material. Also, this method does not present great feasibility for a large amount of shredded material.

In conclusion, after reviewing the investigated methods, it can be seen that mechanical methods have an advantage over other methods because they use chemicals in terms of waste management and the removal of toxic and dangerous organic substances. While they often rely on friction, grinding, or impact, one of the challenges is balancing the temperature control and impact to ensure the substrate (the thermoplastic material) is not damaged. This leads to the potential for innovation in refining temperature and friction settings to optimize efficiency while minimizing substrate degradation. Moreover, developing new mechanical systems that combine friction and minimal heat could significantly reduce paint adhesion without the need for extensive cooling or additional chemical treatments. Investigating systems that utilize low levels of heat with mechanical forces could be a way to minimize both energy consumption and substrate damage during the paint removal process.

On the other hand, solvents do not require machinery. Developments in this paint stripping approach allow other procedures that are much more adjusted to each situation at hand. In addition, although they are generally more effective techniques, they present environmental and operator problems. There is potential in researching ionic liquids, surfactants, and enzyme-based solvents, which might offer lower toxicity and better paint selectivity.

Studies on thermal paint removal highlight its use in contactless surface cleaning. The combination of heat and vibration has shown good results in separating paint layers on thermoplastic surfaces without damaging the base material. However, the economic cost is very high.

One of the crucial goals in recycling painted automotive plastics is to improve the recyclability of materials without compromising quality. Mechanical methods offer advantages by preserving more of the material’s original properties. However, methods like cryogenic and laser-based paint removal might provide cleaner substrates, leading to higher material value and applicability in high-demand industries like automotive manufacturing. The balance between operational costs and improving the recyclability of the final product should be a focus of future research, particularly when it comes to developing industry standards for recycled materials.

In conclusion, an effective paint removal procedure for these thermoplastics would involve a combination of friction, agitation, and temperature, with careful control of these variables throughout the process. Procedures proposed by Nitta et al. [63] and Mizutani et al. [64] could be adapted to this type of methodology. This approach not only eliminates the need for external chemical substances but also aligns with the objectives of the circular economy in plastics, as it avoids the consumption of new material resources during recycling.

## 8. Conclusions

This review examined various methods for recycling polyolefins and removing paint present on their surface in an attempt to search for a better solution to the problem. To achieve high maturity in the recycling industry, the demand for this material must increase and thus achieve a higher presence in the development of improvements and innovations that make the recycling process optimal. For this reason, social awareness and environmental responsibility are as important as the implementation of legislation that regulates both the use of recycled material and the recycling of plastic waste. To advance in the direction of a circular economy, it is necessary to demonstrate that scientific and technological advances can make it possible to obtain recycled materials of good quality. This demonstration would remove the stigma of reusing raw materials as a menial item. In addition, it should be noted that the increase in plastic recovery yields translates into higher profitability and lower raw material costs in manufacturing in the automotive sector. However, passenger safety must be prioritized by ensuring the recycled materials exhibit adequate mechanical properties.

## Figures and Tables

**Table 2:** Paint removal technology list and classification.

Method	Capacity	Effectiveness	Substrate Damage	Environmental Hazard	Economic Balance
*Filtration melt*	✘ ^a^	⭘ ^b^	✔ ^c^	✔	⭘
*High-temperature hydrolysis*	✔	⭘	✔	✔	✔
*Abrasive organic solvents/chemicals*	✔	✔	✘	✘	⭘
*Friction*	✔	⭘	✔	✔	✔
*Blasting*	✘	⭘	⭘	⭘	⭘
*Laser*	⭘	✔	✔	✔	✘

^a^ ✘: negative or nonexistent effect; ^b^ ⭘: intermediate effect or result depends on several factors; ^c^ ✔: positive or significant effect.

## Data Availability

The original contributions presented in the study are available on demand by asking to the corresponding author.
